# Relationship between SNPs of *POU1F1* Gene and Litter Size and Growth Traits in Shaanbei White Cashmere Goats

**DOI:** 10.3390/ani9030114

**Published:** 2019-03-25

**Authors:** Haijing Zhu, Yanghai Zhang, Yangyang Bai, Han Yang, Hailong Yan, Jinwang Liu, Lei Shi, Xiaoyue Song, Longping Li, Shuwei Dong, Chuanying Pan, Xianyong Lan, Lei Qu

**Affiliations:** 1 Shaanxi Provincial Engineering and Technology Research Center of Cashmere Goats, Yulin University, Yulin 719000, China; haijingzhu@yulinu.edu.cn (H.Z.); bai345@126.com (Y.B.); ylhailong@126.com (H.Y.); ljw_yl@163.com (J.L.); shilei_ylxy@126.com (L.S.); psjafh@126.com (X.S.); llp_315@163.com (L.L.); dongshuwei2005@126.com (S.D.); 2 Life Science Research Center, Yulin University, Yulin 719000, China; 3 Key Laboratory of Animal Genetics, Breeding and Reproduction of Shaanxi Province, College of Animal Science and Technology, Northwest A&F University, Yangling 712100, China; yhzhang1997@163.com (Y.Z.); yanghan170902@126.com (H.Y.); chuanyingpan@126.com (C.P.)

**Keywords:** goat, POU class 1 homeobox 1 gene, single nucleotide polymorphisms, diplotype, reproduction, growth traits

## Abstract

**Simple Summary:**

POU (Pit-Oct-Unc) class 1 homeobox 1 (*POU1F1*, or *Pit-1*) is a key transcription factor that directly regulates pituitary hormone-related genes and was considered as a candidate gene for reproduction and growth traits in goat breeding. Three important single nucleotide polymorphisms (SNPs), c.682G > T, c.723T > G and c.837T > C, were found in Shaanbei white cashmere (SBWC) goats by direct DNA sequencing, and c.876 + 110T > C was monomorphic. Further analyses showed that the c.682G > T and c.837T > C loci were associated with litter size, and c.682G > T, c.723T > G and c.837T > C strongly affected growth traits (*p* < 0.05). It is an interesting phenomenon for this population that individuals with the same genotypes had more offspring and that their body status was also better. Thus, we speculated that there was a positive correlation between growth and lambing ability. Besides this, female goats with H3H7 (combined from haplotype 3 and 7) diplotype (GTTT-TTTT), which was consistent with the combination of the optimal genotype of four SNPs, had a better litter size and growth status than other diplotypes. This study provides the first association analyses between the SNPs of *POU1F1* and economic traits in SBWC goats, which could be considered as effective SNP markers for genomic selection in goat breeding.

**Abstract:**

POU (Pit-Oct-Unc) class 1 homeobox 1 (*POU1F1*, or *Pit-1*) is a transcription factor that directly regulates pituitary hormone-related genes, as well as affects the reproduction and growth in mammals. Thus, *POU1F1* gene was investigated as a candidate gene for litter size and growth performance in goats. In the current study, using direct DNA sequencing, c.682G > T, c.723T > G and c.837T > C loci were genotyped in Shaanbei white cashmere (SBWC) goats (n = 609), but c.876 + 110T > C was monomorphic. Besides, the c.682G > T locus was first identified by *Hinf*I (*Haemophilus influenzae* Rf) restriction endonuclease. Association analysis results showed that the c.682G > T, c.837T > C loci and diplotypes were significantly associated with goat litter size (*p* < 0.05). The positive genotypes were GT and TT for the two SNPs, respectively, and the optimal diplotype was H3H7 (GTTT-TTTT). On the other hand, the c.682G > T, c.723T > G and c.837T > C strongly affected growth traits and body measurement indexes in SBWC goats (*p* < 0.05). The positive genotypes or allele of these SNPs were GT, G and TT, respectively. Additionally, the goats with H3H7 diplotype also had a greater growth status than others (*p* < 0.05). Here, individuals with same genotype had both a better litter size and growth traits, showing a positive correlation between these economic traits. Meanwhile, the positive genotypes of four SNPs were combined to obtain the optimal diplotype, which was also H3H7. These SNPs, especially the diplotype, could be used for the genomic selection of excellent individuals with a greater litter size and better growth status in goat breeding.

## 1. Introduction

As one of the earliest domesticated animals, goats play a crucial part in animal husbandry due to their extensive applications, such as in meat, cashmere, or milk production [1]. In order to meet people’s needs, high-production breeds need be selected and raised. However, traditional methods are inefficient, because farms only use phenotypic observations and most economic traits belong to quantitative characters with low heritability. Nowadays, genomic selection (GS) is based on the usage of a set of single nucleotide polymorphisms (SNPs) markers covering the whole genome to estimate genomic estimated breeding value (GEBV) [2,3]. In these cases, some mathematical models were proposed to improve the accuracy of GEBV, such as Bayes or genomic best linear unbiased prediction (GBLUP), which is more accurate and efficient for predicting a specific trait of animals [2,3,4,5]. Therefore, more effective and crucial SNP markers need to be found to make high-density SNP chips and predict breeding values for goat breeding. 

POU (Pit-Oct-Unc) class 1 homeobox 1 (*POU1F1*, initially named *Pit-1*) is a transcription factor belonging to the POU homeodomain family which is characterized by two highly conserved domains, the POU-specific domain and POU-Homeo domain, to bind DNA promoter regions and interact with transcriptional cofactors [6]. *POU1F1* is involved in the development of the anterior pituitary gland and acts as a positive factor for growth hormone (*GH*), prolactin (*PRL*), and thyroid-stimulating hormone beta subunit (*TSHβ*) [7]. The earliest study about natural mutants of *POU1F1* in mice uncovered that the genetic variation of this crucial transcription factor disturbed the expression of three pituitary hormones (GH, PRL, TSH) and led to the phenotype of Snell and Jackson dwarves [8]. Similarly, mutations of *POU1F1* genes resulted in human combined pituitary hormone deficiency which damaged production of pituitary hormones and impaired the growth and development of individuals [9]. In addition, *POU1F1* polymorphisms were significantly associated with porcine growth and meat traits [10,11]; bovine production traits and milk traits [12,13]; ovine milk production and weaning weight traits [14,15]; and goat growth, milk, and cashmere traits [16,17,18]. These potent evidences indicated that the *POU1F1* gene can act as a candidate gene to widely enhance reproduction, growth, meat, milk, and cashmere traits in goat breeding.

Several SNPs located in the *POU1F1* gene have been reported to be associated with economic traits in goats. We previously discovered *POU1F1* gene polymorphisms and analyzed their relationship with production traits in some indigenous Chinese goats [17,18,19,20,21]. We first found that c.723T > G (p.S241S, *Dde*I (*Escherichia coli* carrying the plasmid encoding *DdeI* gene)), c.837T > C (p.S279S, *Alu*I (*Arthrobacter luteus*)), c.876 + 110T > C (3′UTR 110 T > C, *Pst*I (*Escherichia coli* ED8654 carrying the plasmid encoding *PstI* gene)), and 12 other SNPs of *POU1F1* significantly affected milk yield, reproduction, body traits, and cashmere yield. After that, Feng et al. verified c.723T > G and c.837T > C loci in Jining Grey goats, as well as finding a new missense mutation (c.682G > T, p.A228S) indicating that genotype GT individuals had a greater litter size than those with genotype GG [22]. Subsequently, these SNPs were associated with milk performance in Sarda goats [23] and Guanzhong dairy goats [24]. So far, only two studies have detected the SNP polymorphisms of *POU1F1* in Shaanbei white cashmere (SBWC) goats, but the relationship between these polymorphisms and economic traits was not analyzed [25,26]. As an important local breed in Northern China, the SBWC goat is not only an excellent cashmere–meat dual purpose goat, but also has the potential to produce multiple lambs (litter size rate: 105%–283%) [27]. Therefore, exploring potential SNP markers to increase the number of offspring and enhance the growth traits in the SBWC breed is worthy of in-depth study.

In the present study, we reported several significant SNPs in exon 6 and 3′UTR of *POU1F1* gene in SBWC goats, and investigated the association with these SNPs and litter size and growth traits. These data would contribute to the GS method to select a herd with high productivity in the goat breeding.

## 2. Materials and Methods

### 2.1. Animal, Data Collection, and DNA Extraction

All animal experiments adhered to the relevant laws and institutional guidelines and were approved by the Institutional Animal Care and Use Committee of the Yulin University (Approval Number: YLU-2019-02-001). A total of 609 female SBWC goats were selected randomly from the SBWC goat breeding farm [27,28]. These goats were reared on the same farm under similar environmental conditions. We recorded the first-born litter size and measured 9 kinds of growth traits (cm) in these female goats, including body height (BH), body weight (BW), body length (BL), height at hip cross (HHC), chest circumference (ChC), cannon circumference (CC), chest depth (ChD), chest width (ChW), and hip width (HW). Subsequently, corresponding body measurement indexes (%) were calculated, including body index (BI), BL index (BLI), ChC index (ChCI), ChW index (ChWI), CC index (CCI), and limb length index (LLI) ((BH-ChD)/BH) [29]. Then, genomic DNA was extracted from ear tissues of female goats [17]. 

### 2.2. Primer Design, Amplification, and SNPs Scanning

Based on the goat *POU1F1* genomic reference sequence (NC_030808.1) and previous reporting [17], one pair of primers P1 (forward: 5′-CGATCATCTCCCTTCTT-3′ and reverse: 5′-AATGTACAATATGCCTTCTGAG-3′) were designed to amplify a 450 bp product covering exon 6 and 3′UTR. Each PCR reaction (25 μL) contained 1 μL DNA (10 ng/μL), 0.5 μL of each primer (10 μM/μL), 12.5 μL 2 × MIX (Tsingke, Xi’an, China), and 10.5 μL ddH_2_O. The PCR program was as follows: 94 °C for 5 min; 35 cycles of 30 s at 94 °C, 30 s at 54.5 °C, and 30 s at 72 °C; and a final extension at 72 °C for 10 min. All PCR products were verified by 1.5% agarose gel electrophoresis and then directly sequenced by the Tsingke company. Sequence alignment and genotyping were conducted by BioXM 2.6.0 (College of Agriculture, Nanjing Agricultural University, Nanjing, China) and Chromas 2.6.5 (Technelysium Pty Ltd, South Brisbane, Queensland, Australia).

### 2.3. Identification of c.682G > T Locus by HinfI Restriction Endonuclease 

Based on the sequencing results above, c.682G > T locus could be identified by *Hinf*I (*Haemophilus influenzae* Rf) restriction endonuclease if the locus has mutated to T allele (GANTC). Here, a pair of primers P2 were designed as follows, forward: 5′-AGGAGCCTACATGAGACAAGC-3′ and reverse: 5′- CCCGTTTTTCTCTCTGTCTTCG-3′. Each PCR reaction was the same as the previous one. The PCR program was touch down PCR, as follows: 94 °C for 5 min; 18 cycles of 30 s at 94 °C, 30 s at 68 °C (with a decrease of 2–3 °C every two cycle), and 30 s at 72 °C; 30 cycles of 30 s at 94 °C, 30 s at 50 °C, and 30 s at 72 °C; and a final extension at 72 °C for 10 min. A total of 30 samples were selected and verified by endonuclease digestion experiment. The PCR products were verified by 1.5% agarose gel electrophoresis and sequenced. Then, the 5 μL PCR products were digested with 0.5 μL (5 U) QuickCut *Hinf*I (TAKARA, Dalian, China), 1 μL 10× QuickCut Buffer and 3.5 μL ddH_2_O in 5 min at 37 °C. The digested products were directly detected by 3.5% agarose gel electrophoresis. 

### 2.4. Statistical Analysis

The genotypic, allelic frequencies and Hardy–Weinberg equilibrium (HWE) were directly calculated. Population indexes (polymorphism information content, PIC; homozygosity, Ho; heterozygosity, He; effective allele numbers, Ne) were calculated by PopGene version 1.3.1 (Molecular Biology and Biotechnology Centre, University of Alberta, Edmonton, Canada) [30]. These SNPs of *POU1F1* were subjected to linkage disequilibrium (LD) analysis and haplotype analysis by using the SHEsis platform (http://analysis.bio-x.cn/myAnalysis.php) [31,32]. 

Associations between SNPs with litter size were analyzed using the general linear model (GLM): Y_ij_ = μ + HYS_i_ + G_j_ + ε_ij_. In the formula, Y_i_ is the observation of litter size, μ is the population mean, HYS_i_ is the fixed effect of the herd-year-season, G_j_ is the fixed effect of genotype or diplotype, and ε_i_ is the random error [33]. The litter size in this experiment was the first-born litter size; thus, the lambing year and parity had no significant effects on the GLM [33]. In addition, another GLM formula, Y_ik_ = μ + A_i_ + G_k_ + ε_ik_, was performed for the association between SNPs with growth traits. In this formula, Y_i_ is the observation of growth traits, μ is the population mean, A_i_ is the fixed effect of age, G_j_ is the fixed effect of genotype or diplotype, and ε_i_ is the random error [34,35]. One-way ANOVA was used to test the hypothesis that several means are equal and the following multiple test correction (*p*-value correction) was conducted by the Bonferroni method using IBM SPSS Statistics 23.0 (IBM, New York, NY, USA) [36]. A Chi-square test was conducted to test whether there was a significant difference in the different genotypes or diplotypes between the two types of litter size (mothers of a single lamb and multi-lamb) (IBM SPSS Statistics 23.0). The mean ± standard error (S.E.) were denoted for all data and *p* < 0.05 was considered to be a significant difference.

## 3. Results

### 3.1. Identification of POU1F1 Gene Polymorphisms

**Figure 1:**
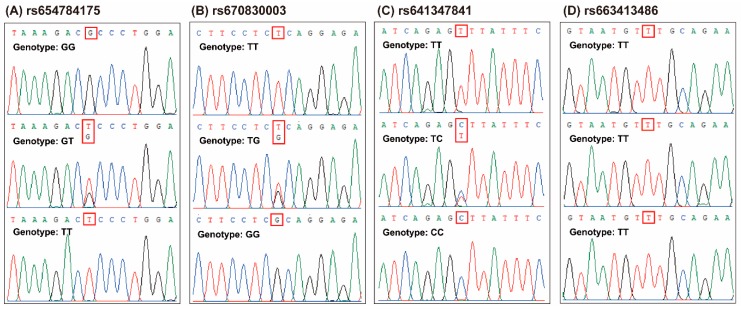
Sequence chromatograms of four single nucleotide polymorphisms (SNPs) in goat POU class 1 homeobox 1 (*POU1F1*) gene ran on Chromas. (**A**) rs654784175 (c.682G > T, p.A228S). (**B**) rs670830003 (c.723T > G, p.S241S). (**C**) rs641347841 (c.837T > C, p.S279S). (**D**) rs663413486 (c.876 + 110T > C, 3′UTR 110 T > C).

In the present study, four SNPs of *POU1F1* gene, c.682G > T, c.723T > G, c.837T > C, and c.876 + 110T > C, were identified in SBWC goats by direct DNA sequencing (Figure 1 and Supplementary Figure S1). The c.682G > T, c.723T > G and c.837T > C loci of *POU1F1* had three genotypes in this population, which were GG/GT/TT, TT/TG/GG, and TT/TC/CC, respectively. However, at the c.876 + 110T > C locus, the SBWC population were monomorphic (Figure 1). The mutated bases of HGVS names were complementary with these in other names because goat *POU1F1* gene is reversely encoded on the chromosome (Table 1). Here, we used a format such as c.682G > T to represent different SNPs in the whole article, whose mutated bases are consistent with previous reports and the results of the current study.

**Figure 2:**
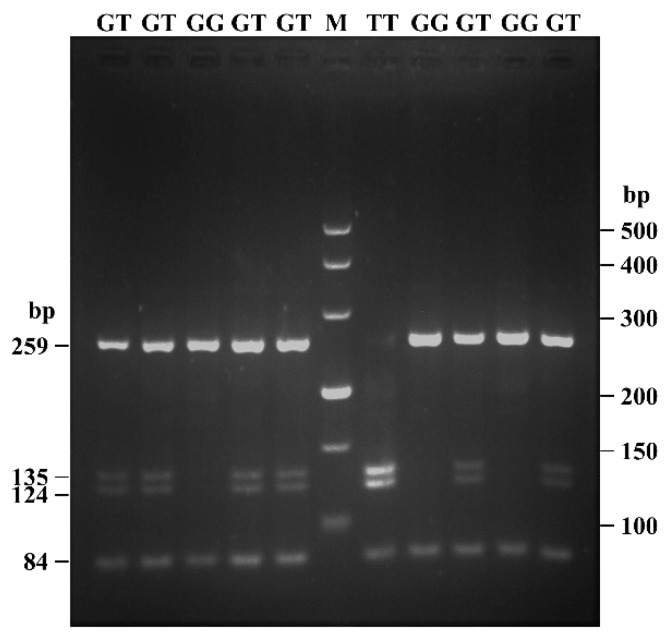
The electrophoresis pattern of c.682G > T locus within goat POU class 1 homeobox 1 (*POU1F1*) digested by *Hinf*I (*Haemophilus influenzae* Rf) endonuclease. “M” represented DNA marker (500 bp, 400 bp, 300 bp, 200 bp, 150 bp, and 100 bp); GT genotype had four bands (84 bp, 124 bp, 135 bp, and 259 bp), GG genotype had two bands (84 bp and 259 bp), and TT genotype showed three bands (84 bp, 124 bp, and 135 bp), respectively.

In addition, to verify whether c.682G > T locus was identified by *Hinf*I restriction endonuclease, a 343 bp DNA fragment was amplified by P2 primer and sequenced. Since another natural recognition sequence (GAGTC) exists on this sequence (Supplementary Figure S2), two fragments (84 bp and 259 bp) represented GG genotype, four fragments (84 bp, 124 bp, 135 bp, and 259 bp) represented GT genotype, and three fragments (84 bp, 124 bp, and 135 bp) were generated from TT genotype after the *Hinf*I digestion (Figure 2). Besides, it was different from the sequence of *Hinf*I locus of *POU1F1* on cattle [37].

### 3.2. Genetic Parameter of POU1F1 Gene Polymorphisms

The genotypic and allelic frequencies of four SNPs were calculated and are shown in Table 2. Briefly, the minor allele frequencies of c.682G > T, c.723T > G, and c.837T > C were 0.062, 0.223, and 0.140, respectively. The c.876 + 110T > C locus was monomorphic in all tested goats, so the minor allele frequency was 0. 

Subsequently, the population indexes of these SNPs were evaluated based the genotypic frequency number, including Ho, He, Ne, and PIC (Table 2). The values of Ho, He, and Ne reflect the degree of genetic variation in this population, and PIC represents the genetic information content. Therefore, the polymorphism of the three genotyped SNPs in this population was not high; the maximum He was 0.358, and the maximum PIC was 0.294, which belonged to the intermediate PIC (0.25 < PIC < 0.50). Moreover, genotypic frequencies of c.682G > T and c.723T > G loci were found in accordance with the HWE (*p* > 0.05), while the c.837T > C locus did not conform to HWE (*p* < 0.05) in the SBWC goat population (Table 2).

### 3.3. LD and Haplotype Analysis

**Figure 3:**
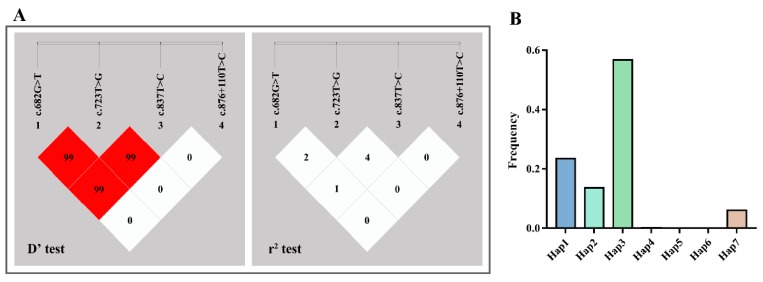
Linkage disequilibrium analysis and haplotype frequency analysis. (**A**) Four loci of the POU class 1 homeobox 1 (*POU1F1*) gene chosen for linkage disequilibrium (LD) analysis in studied populations: locus 1, c.682G > T; locus 2, c.723T > G; locus 3, c.837T > C; locus 4, c.876 + 110T > C. (**B**) The frequencies of seven different haplotypes of four loci in *POU1F1* gene. Hap1: GGTT; Hap2: GTCT; Hap3: GTTT; Hap4: TGCT; Hap5: TGTT; Hap6: TTCT; Hap7: TTTT.

According to the D’ test and r^2^ test in LD analysis, there was linkage equilibrium among four SNPs (Figure 3A and Table 3). The haplotype analysis was carried out by the SHEsis platform and seven different haplotypes of *POU1F1* were found, which were Hap1 (GGTT), Hap2 (GTCT), Hap3 (GTTT), Hap4 (TGCT), Hap5 (TGTT), Hap6 (TTCT), and Hap7 (TTTT) (Figure 3B and Table 4). The highest haplotype frequency was Hap3, which accounted for 56.7% of all haplotypes. Hap1 was the next largest proportion, at 23.5%, followed closely by Hap2 (13.6%) and Hap7 (6.1%). Since haplotypes with frequencies below 5% were meaningless in statistical analysis, haplotypes 4, 5, and 6 were not subjected to subsequent analysis (Table 4).

### 3.4. Association between SNPs of POU1F1 and Litter Size

Association analysis results showed that c.682G > T and c.837T > C loci were significantly associated with first-born litter size in SBWC goats (Table 5). For the c.837T > C locus, female goats with TT genotype had a greater litter size than with other genotypes (*p* = 0.001). However, the relationship between c.682G > T and c.723T > G and litter size showed no significant difference (*p* ≥ 0.05). 

In addition, the genotype distributions of these loci in all individuals with single lamb and multi-lamb litters (≥2) were investigated using the Chi-square test (Table 6). The results indicated that c.682G > T and c.837T > C had different genotype distributions between different litter types of SBWC goats (*p* < 0.05). However, the c.723T > G genotypes showed no difference in the distribution of this population. These results indicated that the c.682G > T and c.837T > C loci of *POU1F1* affected the litter size in SBWC goats (Table 5 and Table 6).

### 3.5. Association between Diplotypes of POU1F1 and Litter Size

Based on the haplotype analysis results (Figure 3B and Table 4) and the frequencies of the diplotypes, four available diplotypes, H3H1 (GTTT-GGTT), H3H2 (GTTT-GTCT), H3H3 (GTTT-GTTT), and H3H7 (GTTT-TTTT), were used to further evaluate the association with the economic traits of SBWC goats. The association analysis showed there was a strong relationship between different diplotypes and litter size (Table 7). Female goats with H3H7 diplotypes had more lambs than other diplotypes (*p* ≤ 0.039). Likewise, the genotype distributions of these diplotypes with different types of litter size were analyzed (Table 8). The result demonstrated that there were different distributions between the two subgroups with different litter size in SBWC goats (*p* = 8.17 × 10^−12^). This result was consistent with the association analysis in Table 7.

### 3.6. SNPs and Diplotypes of POU1F1 Associated with Growth Parameters

The relationship between the SNPs and diplotypes of *POU1F1* gene and growth parameters in SBWC goats were analyzed (Table 9 and Table 10; insignificant data not shown). For the c.682G > T locus, GT genotype female goats laid significantly greatest at BH, HHC, BL, CC, ChW, and ChWI than with other genotypes (*p* < 0.05). At the c.723T > G locus, TG genotype goats had greater BI, ChCI, and CCI than TT genotypes (*p* < 0.05), and GG genotype goats had greater HW than other genotypes (*p* = 0.024). In addition, the TT genotype of the c.837T > C locus individuals had greater HHC, ChC, CC, ChD, ChW, BI, and CCI than those of other genotypes (*p* < 0.05) (Table 9). 

Furthermore, four diplotypes were remarkably associated with growth parameters (Table 10). H3H7 diplotype goats had higher BH, HHC, BL, CC, ChD, ChW, and CCI than other diplotypes (*p* < 0.05), while H3H1 diplotype goats had greater ChC, BI, and ChCI than other diplotypes (*p* < 0.05).

## 4. Discussion

The *POU1F1* gene is the key driver of the expression of *GH*, *PRL*, and *TSHβ* genes and regulates the normal development of the pituitary and growth [7]. Several studies have highlighted *POU1F1* as a candidate gene for reproduction, growth, milk, and cashmere traits in goats [16,17,18,19,20]. However, no studies have analyzed the relationship between SNPs and economic traits in SBWC goats. In this study, the SNPs within exon 6 and 3′UTR of *POU1F1* were identified in SBWC goats (n = 609) via direct DNA sequencing and association, with both litter size and growth traits being investigated.

Based on previous studies, four known SNPs, c.682G > T (p.A228S), c.723T > G (p.S241S; *Dde*I), c.837T > C (p.S279S; *Alu*I), and c.876 + 110T > C (3′UTR 110T > C; *Pst*I), were particularly focused upon in this study (Table 1 and Supplementary Figure S1). The sequence alignment results demonstrated that c.682G > T, c.723T > G, and c.837T > C loci had three genotypes, but the c.876 + 110T > C locus was monomorphic in the SBWC goats (Figure 1). Particularly, c.682G > T locus was first identified by *Hinf*I restriction endonuclease in the current study (Figure 2), which may be conveniently and cheaply used to identified this locus in other goats. 

For c.682G > T locus, the frequency of the mutant allele “T” was very low (0.062) in this population, which was consistent with other breeds of goats (Supplementary Table S1) [22,23]. For example, in Jining Grey, Liaoning Cashmere, and Sarda goats, the frequencies of the allele “T” were also low, which were 0.043, 0.125, and 0.159, respectively; however, this “T” mutation was not found in Guizhou White, Boer, and Wendeng Dairy goats [22,23]. These results indicated that the frequencies of this SNP were not high, which is perhaps due to natural selection and evolution in goats. In 19 breeds of goats, including SBWC goats, the mutant allele “G” frequencies of c.723T > G range from 0 to 0.755 (Supplementary Table S1) [19,21,22,23,24,26], which means the mutation at this locus varies greatly among different breeds. Furthermore, the range of “C” allele frequencies of c.837T > C is still relatively large (0 to 0.523) (Supplementary Table S1) [17,22,23,24,25]. Intriguingly, the frequencies of c.837T > C alleles are very similar in SBWC goats between this study and our previous study [25], while female goats with the CC genotype were not found in the previous study; this may be due to the larger population detected in the present experiment. Finally, the c.876 + 110T > C locus was monomorphic in SBWC goats in this study, which may be on account of the odds of mutation not being high, as with the frequencies of the “C” allele in other four breeds as shown in Supplementary Table S1 [18,23,24]. 

To assess the relationship between the *POU1F1* polymorphisms and reproduction, the first-born litter sizes of SBWC goats were analyzed. Association analyses (Table 5) and Chi-square test results (Table 6) showed that c.682G > T and c.837T > C loci were significantly associated with litter size (*p* < 0.05). Briefly, female goats with the TT genotype at c.837T > C had a greater litter size than other genotypes (*p* = 0.001) and two types of litter size of female goats were remarkably different in each genotype distribution of c.682G > T and c.837T > C loci, respectively (*p* < 0.05). Thus, GT of the c.682G > T locus and TT of the c.837T > C locus were positive genotypes for litter size in SBWC goats. Moreover, it is well known that c.682G > T, c.723T > G, c.837T > C, and c.876 + 110T > C loci of goat *POU1F1* were identified together in many breeds, and so the linkage relationship was analyzed among these loci in the SBWC breed. Unfortunately, there was linkage equilibrium among the four SNPs (Figure 3A and Table 3). Additionally, four haplotypes, Hap1, 2, 3, and 7, were used to combine diplotypes and associated with those important traits. According to the association analysis results (Table 7) and Chi-square testing (Table 8), female goats with H3H7 diplotype (GTTT-TTTT) showed the greatest litter size in SBWC goats. Notably, this result is consistent with analysis at the single loci above: The optimal GT genotype was at the c.682G > T locus, TT at c.723T > G, TT at c.837T > C, and TT at c.876 + 110T > C combinations. Therefore, these SNPs could be used to select excellent individuals with greater litter sizes, especially with the combined diplotype. 

On the other hand, to determine SNPs associated with growth traits in SBWC goats, the relationships between polymorphisms of *POU1F1* and growth parameters were evaluated (Table 9; Table 10). Female goats with the GT genotype of the c.682G > T locus showed superior growth traits; female goats carrying the G allele of c.723T > G had better growth traits; and individuals with TT genotype in c.837T > C showed higher growth parameters than other genotypes (*p* < 0.05; Table 9). Besides, female goats with the H3H7 diplotype (GTTT-TTTT) showed superior growth status in SBWC goats (Table 10). Theoretically, the positive diplotypes were GTTT-TGTT, GGTT-TTTT, or GGTT-TGTT based on the association analysis between single locus and growth traits; however, the frequency of the diplotypes was too low to analyze and further use in practical breeding. Therefore, H3H7 diplotype (GTTT-TTTT) is the best combination genotype for improving growth traits in SBWC goats.

There was a clearly positive relationship between litter size and growth status in the same genotype goats. To be specific, the GT genotype of the c.682G > T locus and TT genotype of the c.837T > C locus were strongly associated with both better litter sizes and growth traits, as well as the H3H7 diplotype being excellent for all traits in SBWC goats in this study. Previous studies indicated that livestock who had more offspring had better body condition [34,38,39], and we believed that this phenomenon also could happen in this population, and that these SNPs may primarily affect the growth and development of goats and then enhance fecundity. 

At present, only two studies reported the relationship among these SNPs of the *POU1F1* gene and litter size in goats (Supplementary Table S1) [17,22]. In the study of Feng et al., they first found that the GT type of the c.682G > T locus was the dominant genotype on the Jining Grey goat [22], while the study of Lan et al. showed that the c.837T > C locus did not affect the litter size [17]. However, in this study, we confirmed that both of them affected litter size in SBWC goats. For growth status, only two articles analyzed the growth-related traits of goats, including the association between the c.837T > C and birth weight, one-year-old weight, and two-year-old weight [17], as well as the relationship between the c.723T > G locus and birth weight, 9-month-old weight, 1-year-old weight, stature, body size, heart girth, and shank girth [21]. In the present study, we measured nine kinds of growth traits and calculated six kinds of corresponding body measurement indexes in adult goats and could comprehensively analyze the correlation between loci and growth status. Actually, most studies have focused on the analysis of the relationship between polymorphisms and milk or cashmere yields, such as milk yield, milk fat, milk protein, hair length, and hair production (Supplementary Table S1) [17,18,19,23,24]. Therefore, whether *POU1F1* gene polymorphisms affect the milk production and cashmere traits of SBWC goats needs further study.

## 5. Conclusions

The c.682G > T, c.723T > G, c.837T > C, and c.876 + 110T > C loci of *POU1F1* gene were identified in SBWC goats, and the first three SNPs were genotyped. Additionally, the c.682G > T locus was first identified by *Hinf*I restriction endonuclease, which may be used to conveniently and cheaply identified this locus in other goats. Analysis results revealed that the c.682G > T and c.837T > C loci were associated with litter size, and c.682G > T, c.723T > G, and c.837T > C strongly affected growth traits. Besides, female goats with H3H7 diplotype (GTTT-TTTT) had a better litter size and growth status than other diplotypes. This study suggests that these SNPs can be considered as effective SNP markers for genomic selection in goat breeding.

## Figures and Tables

**Table 1:** Name information of single nucleotide polymorphisms (SNPs) of the POU class 1 homeobox 1 (*POU1F1*) gene in goats.

refSNP No.	HGVS Names	Other Names				Reference
rs654784175	NC_030808.1:g.34236325C > A	c.682G > T	p.A228S	Ex 6 17 G > T	*Hinf*I *	[19]
rs670830003	NC_030808.1:g.34236284A > C	c.723T > G	p.S241S	Ex 6 58 T > G	*Dde*I	[16]
rs641347841	NC_030808.1:g.34236170A > G	c.837T > C	p.S279S	Ex 6 172 T > C	*Alu*I	[14]
rs663413486	NC_030808.1:g.34236021A > G	c.876 + 110T > C		3′UTR 110 T > C	*Pst*I	[15]

Note: HGVS, Human Genome Variation Society. * The restriction endonuclease site (GANTC) was verified in the present study. The refSNP No. and HGVS names from Ensembl database (http://asia.ensembl.org/index.html).

**Table 2:** Genotypic and allelic frequencies and population indexes for four single nucleotide polymorphisms (SNPs) of the goat POU class 1 homeobox 1 (*POU1F1*) gene in Shaanbei white cashmere (SBWC) goats.

Loci Names	Observed Genotypes (N)	Genotype Frequencies	Allele Frequencies	Ho	He	Ne	PIC	χ2
								(*p* Value)
c.682G > T	GG (526)	0.884	0.938 (G)	0.883	0.117	1.132	0.110	3.600 (*p* = 0.058)
	GT (64)	0.108	0.062 (T)					
	TT (5)	0.008						
c.723T > G	TT (354)	0.581	0.767 (T)	0.642	0.358	1.557	0.294	0.868 (*p* = 0.352)
	TG (226)	0.371	0.233 (G)					
	GG (29)	0.048						
c.837T > C	TT (456)	0.750	0.860 (T)	0.759	0.241	1.317	0.212	4.255 (*p* = 0.039)
	TC (134)	0.220	0.140 (C)					
	CC (18)	0.030						
c.876 + 110T > C	TT (609)	1.000	1.000 (T)	1.000	1.000	1.000	1.000	-
	TC (0)	0.000	0.000 (C)					
	CC (0)	0.000						

Note: Ho, homozygosity; He, heterozygosity; Ne, effective allele numbers; PIC, polymorphism information content.

**Table 3:** Linkage disequilibrium parameters (D’ and r^2^) among c.682G > T, c.723T > G, c.837T > C, c.876 + 110T > C loci in Shaanbei white cashmere (SBWC) goats.

D’\r^2^	c.682G > T	c.723T > G	c.837T > C	c.876 + 110T > C
c.682G > T		0.020	0.010	0.000
c.723T > G	0.991		0.049	0.000
c.837T > C	0.990	0.998		0.000
c.876 + 110T > C	0.000	0.000	0.000	

Note: D’ and r^2^ values are shown in the lower and upper triangle of the Table, respectively.

**Table 4:** The frequencies of each haplotype for four loci of goat POU class 1 homeobox 1 (*POU1F1*) gene.

Haplotype	c.682G > T	c.723T > G	c.837T > C	c.876 + 110T > C	Frequencies
Hap1	G	G	T	T	0.235
Hap2	G	T	C	T	0.136
Hap3	G	T	T	T	0.567
Hap4	T	G	C	T	0.001
Hap5	T	G	T	T	3.03 × 10^−4^
Hap6	T	T	C	T	1.60 × 10^−4^
Hap7	T	T	T	T	0.061

**Table 5:** Relationship between single nucleotide polymorphisms (SNPs) of the POU class 1 homeobox 1 (*POU1F1*) gene and litter size in Shaanbei white cashmere (SBWC) goats.

Loci Names	Genotypes (N)			*p* Value
c.682G > T	GG	GT	TT	
	1.27 ± 0.03 (437)	1.49 ± 0.11 (37)	1.00 ± 0.00 (3)	≥0.061
c.723T > G	TT	TG	GG	
	1.32 ± 0.03 (275)	1.25 ± 0.04 (185)	1.21 ± 0.09 (28)	≥0.476
c.837T > C	TT	TC	CC	
	1.34 ^A^ ± 0.03 (360)	1.13 ^B^ ± 0.04 (111)	1.18 ^AB^ ± 0.13 (17)	=0.001

Note: Values with different letters (A, B) within the same row differ significantly at *p* < 0.01.

**Table 6:** Genotype distribution between mothers of single lamb and multi-lamb in Shaanbei white cashmere (SBWC) goats.

Loci Names	Types	Sample Sizes	Genotypes			Independent χ^2^, *p* Value
c.682G > T			GG	GT		
	Mothers of single lamb	354	332	22		χ^2^ = 4.920; *p* = 0.027
	Mothers of multi-lamb (≥2)	120	105	15		
c.723T > G			TT	TG	GG	
	Mothers of single lamb	366	198	145	23	χ^2^ = 3.208; *p* = 0.201
	Mothers of multi-lamb (≥2)	122	77	40	5	
c.837T > C			TT	TC	CC	
	Mothers of single lamb	366	252	99	15	χ^2^ = 18.307; *p* = 1.04 × 10^−4^
	Mothers of multi-lamb (≥2)	122	108	12	2	

Note: The numbers of GG genotype at the c.682G > T locus were less than five, these data were not calculated. *p* < 0.05 or *p* < 0.01 was considered to be a significant difference.

**Table 7:** Diplotypes of the POU class 1 homeobox 1 (*POU1F1*) gene associated with litter size in Shaanbei white cashmere (SBWC) goats.

Trait	H3H1 (N)	H3H2 (N)	H3H3 (N)	H3H7 (N)	*p* Value
Litter size	1.28 ^bc^ ± 0.05 (137)	1.12 ^c^ ± 0.04 (67)	1.40 ^ab^ ± 0.05 (155)	1.62 ^a^ ± 0.15 (21)	≤0.039

Note: Values with different letters (a, b, c) within the same row differ significantly at *p* < 0.05.

**Table 8:** Diplotypes of the POU class 1 homeobox 1 (*POU1F1*) gene associated with litter size in Shaanbei white cashmere (SBWC) goats.

Types	Sample Sizes	Diplotypes				Independentχ^2^, *p* Value
		H3H1	H3H2	H3H3	H3H7	
Mothers of single lamb	273	104	59	100	10	χ^2^ = 54.647;
Mothers of multi-lamb (≥2)	77	33	8	55	11	*p* = 8.17 × 10^−12^

Note: H3H1: GTTT-GGTT; H3H2: GTTT-GTCT; H3H3: GTTT-GTTT; H3H7: GTTT-TTTT. *p* < 0.05 or *p* < 0.01 was considered to be a significant difference.

**Table 9:** Relationship between single nucleotide polymorphisms (SNPs) of the POU class 1 homeobox 1 (*POU1F1*) gene and growth parameters in Shaanbei white cashmere (SBWC) goats.

Loci Names	Parameters	Genotypes (N)			*p* Value
c.682G > T		GG	GT	TT	
	BH (cm)	57.10 ^b^ ± 0.21 (466)	58.67 ^a^ ± 0.59 (57)	57.80 ^ab^ ± 1.71 (5)	=0.044
	HHC (cm)	60.24 ^b^ ± 0.23 (465)	62.27 ^a^ ± 0.64 (57)	59.90 ^ab^ ± 2.06 (5)	=0.011
	BL (cm)	63.27 ^B^ ± 0.26 (466)	65.70 ^A^ ± 0.71 (57)	63.80 ^AB^ ± 1.63 (5)	=0.006
	CC (cm)	7.89 ^b^ ± 0.05 (467)	8.25 ^a^ ± 0.14 (56)	7.40 ^ab^ ± 0.53 (5)	=0.028
	ChW (cm)	18.75 ^B^ ± 0.17 (467)	20.23 ^A^ ± 0.51 (57)	17.90 ^AB^ ± 1.89 (5)	=0.015
	ChWI (%)	68.24 ^B^ ± 0.47 (467)	72.70 ^A^ ± 1.75 (57)	64.26 ^AB^ ± 5.46 (5)	=0.008
c.723T > G		TT	TG	GG	
	HW (cm)	20.98 ^ab^ ± 0.23 (99)	20.56 ^b^ ± 0.40 (53)	23.42 ^a^ ± 0.75 (6)	=0.024
	BI (%)	138.69 ^B^ ± 0.87 (303)	142.94 ^A^ ± 1.12 (202)	141.72 ^AB^ ± 3.66 (28)	=0.009
	ChCI (%)	153.90 ^B^ ± 1.03 (303)	158.98 ^A^ ± 1.31 (203)	157.78 ^AB^ ± 4.50 (27)	=0.008
	CCI (%)	13.71 ^b^ ± 0.10 (306)	14.11 ^a^ ± 0.13 (203)	13.93 ^ab^ ± 0.34 (27)	=0.035
c.837T > C		TT	TC	CC	
	HHC (cm)	60.89 ^A^ ± 0.26 (400)	59.11 ^B^ ± 0.40 (121)	58.29 ^AB^ ± 1.10 (17)	=0.002
	ChC (cm)	89.97 ^A^ ± 0.51 (400)	86.80 ^B^ ± 1.01 (121)	81.33 ^B^ ± 2.33 (15)	≤0.010
	CC (cm)	8.04 ^A^ ± 0.05 (401)	7.60 ^B^ ± 0.08 (121)	7.21 ^B^ ± 0.26 (17)	≤0.002
	ChD (cm)	27.64 ^A^ ± 0.14 (402)	26.75 ^B^ ± 0.25 (121)	27.32 ^AB^ ± 0.66 (17)	=0.007
	ChW (cm)	19.15 ^a^ ± 0.19 (402)	18.06 ^b^ ± 0.31 (121)	17.97 ^ab^ ± 0.71 (17)	=0.015
	BI (%)	141.58 ^a^ ± 0.77 (398)	138.41 ^a^ ± 1.54 (120)	127.21 ^b^ ± 2.83 (15)	≤0.027
	CCI (%)	14.02 ^a^ ± 0.09 (399)	13.50 ^b^ ± 0.16 (120)	13.04 ^ab^ ± 0.43 (17)	=0.012

Note: BH, body height; HHC, height at hip cross; BL, body length; ChC, chest circumference; CC, cannon circumference; ChD, chest depth; ChW chest width; HW, hip width; BI, body index; ChCI, ChC index; ChWI, ChW index; CCI, CC index. Values with different letters (a, b/A, B) within the same row differ significantly at *p* < 0.05/*p* < 0.01.

**Table 10:** Diplotypes of the POU class 1 homeobox 1 (*POU1F1*) gene associated with growth parameters in Shaanbei white cashmere (SBWC) goats.

Parameters	H3H1 (N)	H3H2 (N)	H3H3 (N)	H3H7 (N)	*p* Value
BH (cm)	56.93 ^ab^ ± 0.40 (148)	56.62 ^b^ ± 0.49 (73)	57.96 ^ab^ ± 0.36 (166)	59.21 ^a^ ± 0.76 (33)	=0.048
HHC (cm)	60.51 ^AB^ ± 0.42 (147)	58.81 ^B^ ± 0.49 (73)	61.07 ^A^ ± 0.41 (165)	62.94 ^A^ ± 0.88 (33)	≤0.008
BL (cm)	63.35 ^ab^ ± 0.47 (147)	62.57 ^b^ ± 0.56 (73)	63.84 ^ab^ ± 0.46 (166)	65.94 ^a^ ± 1.02 (33)	=0.029
ChC (cm)	91.16 ^A^ ± 0.80 (147)	86.34 ^B^ ± 1.32 (73)	89.52 ^AB^ ± 0.81 (166)	90.66 ^AB^ ± 1.78 (32)	=0.007
CC (cm)	8.09 ^A^ ± 0.08 (147)	7.51 ^B^ ± 0.10 (73)	8.01 ^A^ ± 0.08 (167)	8.45 ^A^ ± 0.19 (32)	≤0.002
ChD (cm)	27.57 ^ab^ ± 0.23 (147)	26.60 ^b^ ± 0.32 (73)	27.69 ^a^ ± 0.23 (167)	28.32 ^a^ ± 0.53 (33)	≤0.042
ChW(cm)	19.14 ^a^ ± 0.32 (147)	17.50 ^b^ ± 0.31 (73)	19.13 ^a^ ± 0.30 (167)	20.21 ^a^ ± 0.69 (33)	≤0.013
ChCI (%)	160.78 ^a^ ± 1.48 (147)	153.39 ^b^ ± 2.48 (72)	154.95 ^b^ ± 1.30 (165)	153.13 ^ab^ ± 2.78 (32)	=0.026
CCI (%)	14.27 ^A^ ± 0.15 (147)	13.33 ^B^ ± 0.19 (72)	13.86 ^AB^ ± 0.13 (166)	14.28 ^AB^ ± 0.30 (32)	=0.001

**Note:** BH, body height; HHC, height at hip cross; BL, body length; ChC, chest circumference; ChD, chest depth; ChW chest width; ChCI, ChC index; CCI, cannon circumference index. The values with different letters (a, b/A, B) within the same row differ significantly at *p* < 0.05/*p* < 0.01.

## Supplementary Materials

The following are available online at https://www.mdpi.com/2076-2615/9/3/114/s1, Figure S1: Schematic illustration of goat POU class 1 homeobox 1 (*POU1F1*) gene, single nucleotide polymorphisms (SNPs) location, and restriction endonuclease sites, Figure S2: Sequence chromatograms of goat POU class 1 homeobox 1 (*POU1F1*) gene amplified by primer P2, Table S1: The frequencies of c.682G > T, c.723T > G, c.837T > C, and c.876 + 110T > C, and their significant association with economic traits in different goat breeds.
