# Supplementary material for: Relationship between SNPs of POU1F1 Gene and Litter Size and Growth Traits in Shaanbei White Cashmere Goats
Source: Animals (Basel). 2019 Mar 25;9(3):114. doi: 10.3390/ani9030114 (PMC6466355; doi:10.3390/ani9030114)
Supplement: Supplementary file 1 [file animals-09-00114-s001.pdf]

animals

MDPI

Goat *POU1F1* genomic reference sequence (NC_030808.1)

Intron 5

––––TTCCTGTGATTCTGGTAAAAGGAGCCTACATGAGACAAGCATCTAAATGTTCAAA

→ P2 forward primer

AAAAACTTCACATTTATTATTGTTGAAGAGCTTGGAAGGTGTTTGCAGAGTCTAGGTTT

CCTTTTTACGTTAATGCTAATACTAATGTTTAGGAAATTTAACCTAACTTGATTTCGATCA

→ P1 forward primer

Exon 6

TCTCCCTTCTTCTTTCCTGCCAACTCCCCACCTCCCAG|TATTGCTGCTAAAGACGCCCT

c.682G>T

GGAGAGACACTTTGGAGAACAGAATAAGCCTTCCTCTCAGGAGATCCTGAGGATGGC

c.723T>G

TGAAGAACTAAACCTGGAGAAAGAAGTGGTGAGGGTTTGGTTTTGTAACCGAAGACA

GAGAGAAAAACGGGTGAAAACAAGCCTGAATCAGAGTTTATTTCCTATCTCTAAGGAG

← P2 reverse primer c.837T>C

3' UTR

CATCTTGAATGCAGATAG|GTCTCCCATTGTGTAATAGCGAGTTTTTCTGCTTTTCTTTCC

CTTCTCTTCTCCAGCCAAAGTAGAAATCAGTTATTTGGTTAGCTTCCAAACGTCACATC

AGTAATGTTTGCAGAAGTGTTTCTCTTCTACTTTAAAAACAAATACAATTTAAATTATGT

c.876+110T>C

TGATGAATTATTCTCAGAAGGCATATTGTACATTTTAAGCCAAAAACTAATAGGATTAAA

← P1 reverse primer

ACAATGATTCTGTC

Figure S1. Schematic illustration of goat POU class 1 homeobox 1 (*POU1F1)* gene, single nucleotide polymorphisms (SNPs) location, and restriction endonuclease sites. The gray shades represent primers information. The black squares filled with yellow represent four SNPs loci, followed by c.682G>T, c.723T>G, c.837T>C, and c.876+110T>C. The red underlines denote restriction endonuclease site, the order is *Hinf*I, *Dde*I, *Alu*I, and *Pst*I.

animals

MDPI

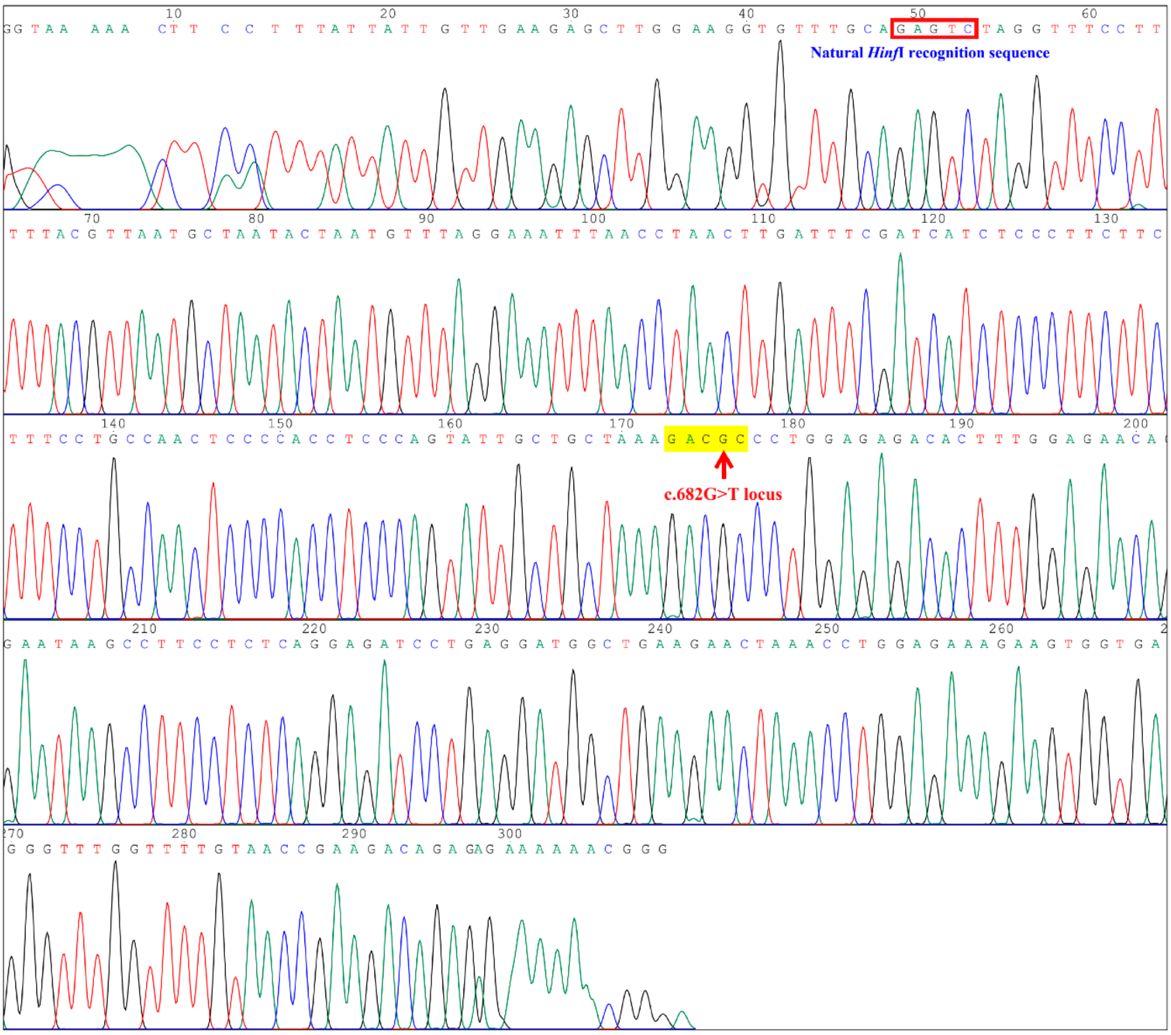

Figure S2. Sequence chromatograms of goat POU class 1 homeobox 1 (*POU1F1*) gene amplified by primer P2. The red arrow points c.682G>T locus and the yellow shade represents *Hinf*I restriction endonuclease site. The red box represents another natural *Hinf*I recognition sequence.

animals

MDPI

Table S1. The frequencies of c.682G>T, c.723T>G, c.837T>C, and c.876+110T>C, and their significant association with economic traits in different goat breeds.

Loci names	Breeds	Sample sizes	Genotype frequencies			Allele frequencies		Significant association (positive genotype)	References
c.682G>T			GG	GT	TT	G	T		
	SBWC	595	0.884	0.108	0.008	0.938	0.062	Litter size (GT); BH, HHC, BL, CC, ChW, ChWI (GT)	In this study
	Jining Grey	163	0.914	0.086	0.000	0.957	0.043	Litter size (GT)	19
	Guizhou White	59	1.000	0.000	0.000	1.000	0.000	-	
	Boer	30	1.000	0.000	0.000	1.000	0.000	-	
	Wendeng Dairy	48	1.000	0.000	0.000	1.000	0.000	-	
	Liaoning Cashmere	40	0.750	0.250	0.000	0.875	0.125	-	
	Sarda	129	0.713	0.256	0.031	0.841	0.159	Milk yield (TT); Fat content (GT, TT)	20
c.723T>G			TT	TG	GG	T	G		
	SBWC	609	0.581	0.371	0.048	0.767	0.233	HW (GG); BI, ChCI, CI (TG)	In this study
	Jining Grey	163	0.632	0.331	0.037	0.798	0.202	No significant	19
	Guizhou White	59	0.712	0.288	0.000	0.856	0.144	-	
	Boer	30	0.734	0.133	0.133	0.800	0.200	-	
	Wendeng Dairy	48	0.854	0.146	0.000	0.927	0.073	-	
	Liaoning Cashmere	40	0.775	0.125	0.100	0.838	0.162	-	
	Sarda	129	0.039	0.411	0.550	0.245	0.755	Milk fat content, milk protein content (TT)	20
	IMWC	452	0.750	0.250	0.000	0.875	0.125	Litter sizes, one-year-old weight (TG)ilk yields (TG)	16;18

	Xinong Saanen dairy	74	0.770	0.230	0.000	0.885	0.115	Milk yields (TG); Litter sizes, one-year-old weight (TG)	
	Laoshan dairy	80	0.200	0.800	0.000	0.600	0.400	Milk yields (TG); Litter sizes, one-year-old weight (TG)	
	Guanzhong dairy	62	0.694	0.306	0.000	0.847	0.153	Milk yields (TG); Litter sizes, one-year-old weight (TG)	
	Guizhou Black	21	1.000	0.000	0.000	1.000	0.000	-	
	Matou	22	0.455	0.545	0.000	0.727	0.273	-	
	Banjiao	25	0.840	0.160	0.000	0.920	0.080	-	
	Guizhou White	31	0.355	0.645	0.000	0.706	0.294	-	
	Leizhou	34	0.418	0.588	0.000	0.777	0.223	-	
	Nanjiang	247	0.324	0.514	0.162	0.581	0.419	-	23
	Xinjiang	175	0.663	0.263	0.074	0.794	0.206	-	
	SBWC*	150	0.460	0.400	0.140	0.660	0.340	-	
	Boer	84	0.774	0.226	0.000	0.887	0.113	-	
	Haimen	33	0.576	0.333	0.091	0.742	0.258	-	
	Xuhuai	20	0.700	0.300	0.000	0.850	0.150	-	
	Guanzhong dairy	235	0.716	0.265	0.019	0.848	0.152	No significant	21
c.837T>C			TT	TC	CC	T	C		
	SBWC	608	0.750	0.220	0.030	0.860	0.140	Litter size, HHC, CC, ChD, ChW, BI, CCI (TT)	In this study
	Jining Grey	178	0.247	0.511	0.242	0.503	0.497	No significant	19
	Guizhou White	58	0.207	0.569	0.224	0.491	0.509	-	
	Boer	30	1.000	0.000	0.000	1.000	0.000	-	
	Wendeng Dairy	47	0.638	0.277	0.085	0.777	0.223	-	
	Liaoning Cashmere	47	0.468	0.468	0.064	0.702	0.298	-	

	Sarda	129	0.891	0.109	0.000	0.945	0.055	No significant	20
	IMWC	452	0.739	0.25	0.011	0.864	0.136	Birth weight (TG)	14
	Xinong Saanen dairy	74	0.946	0.054	0.000	0.973	0.027	Milk yields (TT); birth weight (TG)	
	Laoshan dairy	80	0.513	0.487	0.000	0.756	0.244	Milk yields (TT); birth weight (TG)	
	Guanzhong dairy	62	1.000	0	0.000	1.000	0.000	Milk yields (TT); birth weight (TG)	
	Guizhou Black	21	0.095	0.905	0.000	0.548	0.452	-	
	Matou	22	0.136	0.682	0.182	0.477	0.523	-	
	Banjiao	25	0.160	0.840	0.000	0.580	0.420	-	
	Guizhou White	31	0.226	0.774	0.000	0.613	0.387	-	
	Leizhou	34	0.176	0.824	0.000	0.588	0.412	-	
	SBWC*	213	0.718	0.282	0.000	0.859	0.141	-	22
	Boer	85	0.952	0.048	0.000	0.976	0.024	-	
	Xuhuai	35	0.572	0.371	0.057	0.757	0.243	-	
	Haimen	61	0.820	0.131	0.049	0.885	0.115	-	
	Guanzhong dairy	235	0.953	0.047	0.000	0.977	0.023	Milk performance[†] (TT)	21
c.876+110T>C			TT	TC	CC	T	C		
	SBWC	609	1.000	0.000	0.000	1.000	0.000	-	In this study
	Sarda	129	0.899	0.093	0.008	0.946	0.055	No significant	20
	IMWC	847	0.917	0.083	0.000	0.959	0.041	Cashmere yield[‡] (TT)	15
	Guanzhong dairy	235	0.446	0.524	0.030	0.708	0.292	No significant	21

Note: -, lacking associations analyses between different genotypes and traits in this breed. †, milk performance includes average fat content (%), average acidity, morning milk fat content (%), morning milk acidity, afternoon milk fat content (%) and afternoon milk acidity; ‡, cashmere yield includes two-year-old cashmere yield (g), four-year-old cashmere yield (g), five-year-old cashmere yield (g), average cashmere yield (g). IMWC, Inner Mongolia White Cashmere goat.
